# Antecedent hypertension and myocardial injury in patients with reperfused ST-elevation myocardial infarction

**DOI:** 10.1186/s12968-016-0299-1

**Published:** 2016-11-11

**Authors:** Sebastian J. Reinstadler, Thomas Stiermaier, Charlotte Eitel, Mohammed Saad, Bernhard Metzler, Suzanne de Waha, Georg Fuernau, Steffen Desch, Holger Thiele, Ingo Eitel

**Affiliations:** 1 University Heart Center Lübeck, Medical Clinic II, Department of Cardiology, Angiology and Intensive Care Medicine, University of Lübeck, Ratzeburger Allee 160, 23538 Lübeck, Germany; 2 German Center for Cardiovascular Research (DZHK), Partner Site Hamburg/Kiel/Lübeck, Ratzeburger Allee 160, 23538 Lübeck, Germany; 3 University Clinic of Internal Medicine III, Cardiology and Angiology, Medical University of Innsbruck, Anichstraße 35, A-6020 Innsbruck, Austria

**Keywords:** Hypertension, ST-elevation myocardial infarction, Cardiovascular magnetic resonance, Major adverse cardiac events

## Abstract

**Background:**

Antecedent hypertension is associated with poor outcome in patients with ST-elevation myocardial infarction (STEMI). Whether differences in myocardial salvage, infarct size and microvascular injury contribute to the adverse outcome is unknown. We investigated the association between antecedent hypertension and cardiovascular magnetic resonance (CMR) parameters of myocardial salvage and damage in a multicenter CMR substudy of the AIDA-STEMI trial (Abciximab Intracoronary versus intravenously Drug Application in ST-elevation myocardial infarction).

**Methods:**

We analyzed 792 consecutive STEMI patients reperfused within 12 h after symptom onset. Patients underwent CMR imaging for assessment of myocardial salvage, infarct size and microvascular obstruction within 10 days after infarction. Major adverse cardiac events (MACE) were recorded at 12-month follow-up.

**Results:**

Antecedent hypertension was present in 540 patients (68 %) and was associated with a significantly increased baseline risk profile (advanced age, higher body mass index, higher incidence of diabetes, hypercholesterolemia, previous angioplasty and multivessel disease, *p* < 0.001 for all). MACE were more frequent in patients with hypertension as compared to patients without hypertension (45 [8 %] vs. 8 [3 %], *p* < 0.01). Antecedent hypertension remained an independent predictor of MACE after multivariate adjustment (hazard ratio 3.42 [confidence interval 1.45–8.08], *p* < 0.01). There was, however, no significant difference in the area at risk, infarct size, myocardial salvage index, extent of microvascular obstruction, and left ventricular ejection fraction between the groups (all *p* > 0.05).

**Conclusion:**

Despite a higher rate of MACE in contemporary reperfused STEMI patients with antecedent hypertension, there was no difference in reperfusion efficacy, infarct size and reperfusion injury as visualized by CMR.

**Trial registration:**

NCT00712101.

## Background

Arterial hypertension (HTN) is a major cardiovascular risk factor affecting approximately 30–45 % of the general population [1, 2]. HTN is not only associated with an increased incidence of other risk factors, but also directly contributes to the development and progression of atherosclerotic disease [3]. Accordingly, patients with myocardial infarction (MI) and a history of HTN are known to be older and to have a higher rate of comorbidities and more extensive atherosclerotic disease than patients with MI but no HTN [4]. Several studies demonstrated that patients with MI and antecedent HTN are at increased risk for adverse cardiovascular events, such as heart failure, recurrent MI or death [4–11]. However, whether differences in myocardial salvage, infarct size or microvascular injury contribute to the adverse outcome in contemporary reperfused MI patients is unknown. Cardiovascular magnetic resonance (CMR) is the reference technique for the determination of these infarct characteristics [12], which are known to be strongly correlated with clinical prognosis in patients with ST-elevation MI (STEMI) reperfused by primary percutaneous coronary intervention (PPCI) [13–16].

The aim of this study was therefore to assess the association of antecedent HTN with myocardial salvage and damage determined by CMR as well as to evaluate its prognostic significance on adverse clinical events in a large multicenter cohort of consecutive patients treated with PPCI for STEMI.

## Methods

### Study design and population

This was an analysis of data from the multicenter CMR substudy of the AIDA STEMI trial (Abciximab Intracoronary versus intravenously Drug Application in ST-Elevation Myocardial Infarction; NCT00712101). The rationale, design and main results of AIDA STEMI as well as its CMR substudy have been reported elsewhere [15, 17–20]. Briefly, AIDA STEMI randomized 2065 consecutive STEMI patients treated within 12 h after symptom onset to either intracoronary or intravenous abciximab bolus (0.25 mg/kg) during PPCI with a subsequent 12 h dose-adjusted intravenous infusion. The CMR substudy enrolled 795 patients at 8 sites with proven expertise in CMR imaging, which was performed within 10 days after infarction. Importantly, there was no difference in CMR parameters of myocardial salvage and damage between both treatment groups [20]. The presence of antecedent arterial HTN was prospectively questioned and hypertension was diagnosed if patients were on antihypertensive treatment or had ≥3 systolic blood pressure values >140 mmHg on at least two different days. Patients were categorized as having antecedent HTN or not. Major adverse cardiac events (MACE), defined as a composite of all-cause death, nonfatal re-MI and new congestive heart failure, were recorded at 12 months after STEMI. Detailed endpoint definitions have been published previously [17]. The local ethics committees approved the study and all patients gave a written informed consent.

### Cardiovascular magnetic resonance

Patients were examined at rest in the supine position on 1.5 or 3.0 Tesla scanners at day 1 to 10 after STEMI. Standardized imaging sequences were used as described previously [17, 20, 21]. Blinded readers performed image analysis at the CMR core laboratory (University of Leipzig - Heart Center, Leipzig, Germany). Commercially available software was used for post-processing (cmr42, Circle Cardiovascular Imaging Inc., Calgary, Alberta, Canada). The core laboratory has proven excellent reproducibility and low inter- as well as intra-observer variability in patients with acute STEMI [22]. Measurements of the area-at-risk, infarct size, and microvascular obstruction were expressed as the percentage of left ventricular (LV) volume (%LV). Myocardial salvage index was determined from area at risk and infarct size as previously described [13]. As described previously [20], LV function was available for 792 patients. Late gadolinium enhancement (LGE) for the determination of infarct size and microvascular obstruction was available in 774 patients. T2-weighted imaging for the assessment of the area-at-risk was available for 695 patients. The prevalence of incomplete CMR scans was not significantly different between patients with or without antecedent HTN.

### Statistical analysis

Since most of the continuous variables were non-normally distributed, all continuous data were expressed as median with interquartile range for reasons of uniformity. Categorical data were depicted as counts and percentage. Distribution of data was tested using the Shapiro-Wilk test. Testing for differences between groups was performed with Mann–Whitney-*U* test or Kruskal-Wallis test for continuous variables and *x* ^2^-test for categorical variables. To identify predictors of infarct size, microvascular obstruction, myocardial salvage index, and left ventricular ejection fraction, multiple linear regression analysis was performed. Outcome functions were assessed using Kaplan-Meier graphs with log-rank comparison between groups. In addition, univariate and multivariate Cox regression analysis was performed to identify predictors for time to MACE. Multivariate analysis was performed using variables with a *p* < 0.05 in univariate analysis. All variables listed in Table 1 were assessed in univariate analysis (except the TIMI risk score). Tests were two-tailed, and a probability value of <0.05 was considered statistically significant. All analyses were performed using SPSS version 22.0.0 (IBM, Armonk, NY, USA).

**Table 1:** Baseline characteristics

	All patients (*n* = 792)	Hypertension (*n* = 540)	No hypertension (*n* = 252)	*p* value
Age, years	62 [51–71]	66 [55–73]	54 [48–63]	<0.001
Male sex, n (%)	600 (76)	389 (72)	211 (84)	<0.001
Body mass index, kg/m^2^	27 [25–30]	28 [25–31]	26 [24–29]	<0.001
Cardiovascular risk factors				
Current smoking, n (%)	339/724 (43)	194/482 (36)	145/242 (58)	<0.001
Diabetes mellitus, n (%)	160/789 (20)	142/539 (26)	18/250 (7)	<0.001
Hypercholesterolemia, n (%)	303/784 (38)	250/536 (46)	53/250 (21)	<0.001
Family history for CAD, n (%)	268/611 (34)	177/407 (33)	91/204 (36)	0.79
Systolic blood pressure, mmHg	130 [117–147]	135 [119–150]	126 [114–140]	<0.001
Diastolic blood pressure, mmHg	80 [70–88]	80 [70–90]	80 [70–85]	0.07
Heart rate, beats/min	76 [67–87]	76 [68–88]	76 [65–85]	0.23
Time from symptom onset to PCI hospital admission, min	180 [109–310]	183 [110–311]	166 [104–306]	0.23
Door-to-balloon time, min	30 [22–42]	30 [23–42]	29 [20–42]	0.56
Previous infarction, n (%)	48/791 (6)	42/539 (8)	6/631 (2)	<0.01
Previous PPCI, n (%)	67 (9)	61 (11)	6 (2)	<0.001
Previous CABG, n (%)	11 (1)	11 (2)	0 (0)	0.02
Anterior infarction, n (%)	395/755 (50)	262/516 (49)	133/239 (53)	0.21
Killip-class on admission				0.52
1, n (%)	696 (88)	472 (87)	224 (89)	
2, n (%)	59 (7)	42 (8)	17 (7)	
3, n (%)	20 (3)	16 (3)	4 (2)	
4, n (%)	17 (2)	10 (2)	7 (3)	
Number of diseased vessels				<0.01
1, n (%)	419 (53)	261 (48)	158 (63)	
2, n (%)	225 (28)	165 (31)	60 (24)	
3, n (%)	148 (19)	114 (21)	34 (14)	
Infarct related artery				0.59
Left anterior descending, n (%)	344 (43)	240 (44)	104 (41)	
Right coronary artery, n (%)	344 (43)	226 (42)	118 (47)	
Left circumflex, n (%)	97 (12)	68 (13)	29 (12)	
Left main, n (%)	5 (1)	4 (1)	1 (0)	
Bypass graft, n (%)	2 (0)	2 (0)	0 (0)	
TIMI risk score	3 [2–5]	4 [2–5]	2 [2–4]	<0.001
TIMI-flow before PPCI				0.64
TIMI-flow 0, n (%)	444 (56)	298 (55)	146 (58)	
TIMI-flow 1, n (%)	103 (13)	73 (14)	30 (12)	
TIMI-flow 2, n (%)	128 (16)	92 (17)	36 (14)	
TIMI-flow 3, n (%)	117 (15)	77 (14)	40 (16)	
TIMI-flow after PPCI				0.38
TIMI-flow 0, n (%)	12 (2)	9 (2)	3 (1)	
TIMI-flow 1, n (%)	19 (2)	14 (3)	5 (2)	
TIMI-flow 2, n (%)	61 (8)	47 (9)	14 (6)	
TIMI-flow 3, n (%)	699 (88)	469 (87)	230 (91)	
Stent implanted, n (%)	774 (98)	527 (98)	247 (98)	0.60
Thrombectomy, n (%)	190 (24)	124 (23)	66 (26)	0.32
Concomitant medications				
Aspirin, n (%)	790 (100)	538 (100)	252 (100)	1.0
ß-blockers, n (%)	757 (96)	519 (96)	238 (94)	0.19
ACE-I/ARB, n (%)	751 (95)	515 (95)	236 (94)	0.21
Statin, n (%)	749 (95)	508 (94)	241 (96)	0.47
Aldosterone antagonist, n (%)	80 (10)	55 (12)	25 (10)	0.34

Data are given as median plus interquartile range or number and percentage

*Abbreviations*: *CAD* coronary artery disease, *PCI* percutaneous coronary intervention, *CABG* coronary artery bypass grafting, *TIMI* thrombolysis in myocardial infarction, *ACE-I* angiotensin converting enzyme inhibitor, *ARB* angiotensin receptor blocker

## Results

### Study population

Between July 2008 and April 2011 795 STEMI patients were enrolled in the CMR substudy of the AIDA STEMI trial. Information on antecedent HTN was available in 792 (99.6 %) patients, representing the final study cohort. Characteristics of the overall cohort as well as stratified by history of HTN are listed in Table 1. Antecedent HTN was associated with more advanced age, a higher body mass index, a higher prevalence of female sex, diabetes mellitus, hypercholesterolemia (*p* < 0.001 respectively), multivessel disease (*p* < 0.01), and previous myocardial infarction (*p* < 0.01), but a lower prevalence of current smoking (*p* < 0.001). HTN was also associated with a higher systolic blood pressure on admission (*p* < 0.001) and a trend for a higher diastolic blood pressure (*p* = 0.07) with no significant difference in heart rate (*p* = 0.23). Pre- and post-PPCI TIMI-flow were equal in both groups (*p* = 0.64 and *p* = 0.38, respectively).

### Antecedent hypertension and clinical outcome

**Fig. 1:**
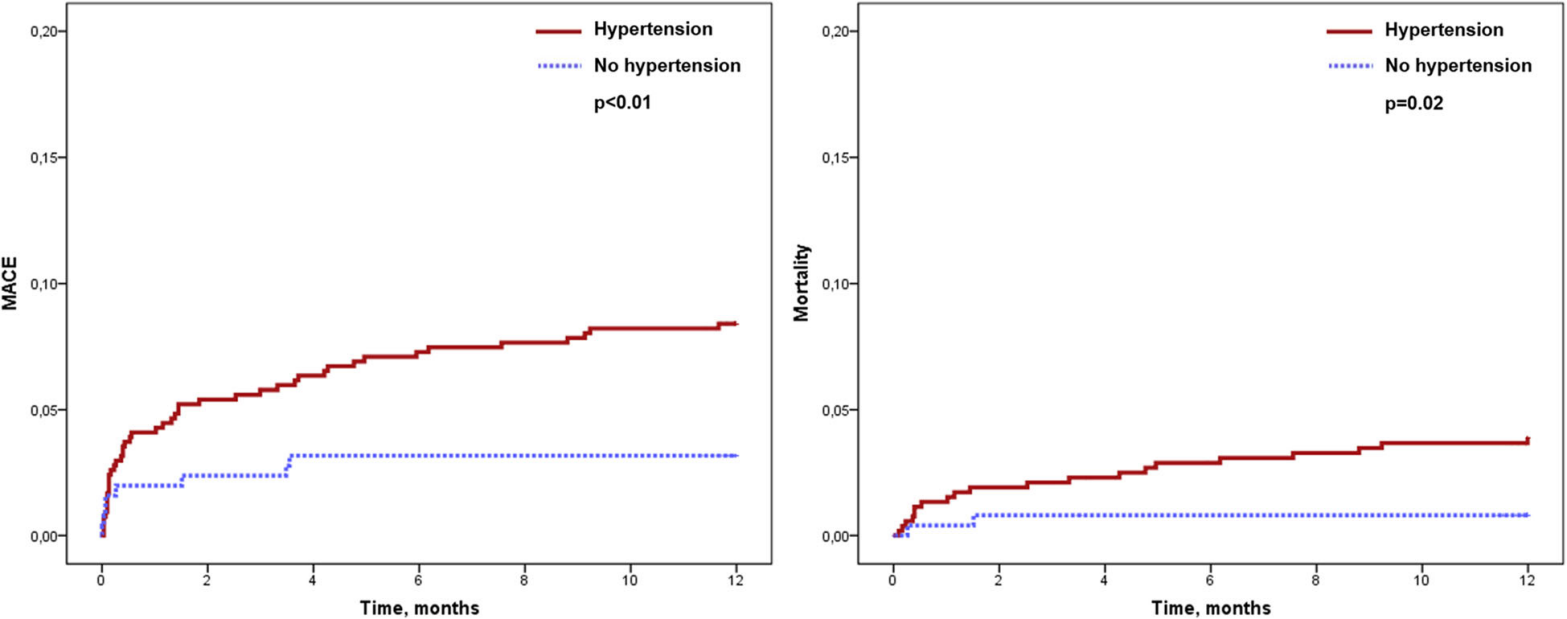
Kaplan-Meier curves for the occurrence of major adverse cardiac events and death according to antecedent hypertension

During the follow-up period of 12 months, 53 patients (7 %) experienced MACE. Of these 22 (3 %) died. Patients with antecedent HTN were at higher risk for MACE (45 [8 %] versus 8 [3 %], *p* < 0.01; hazard ratio: 2.70 [1.27–5.72], *p* = 0.01) and mortality (20 [4 %] versus 2 [1 %], *p* = 0.02; hazard ratio: 4.83 [1.13–20.67], *p* = 0.03) as compared to patients without antecedent HTN (Table 2). Consequently, antecedent HTN showed a significant association with MACE- and mortality-free survival (*p* < 0.01 and *p* = 0.02, respectively, Fig. 1). In multivariate Cox regression analysis, antecedent HTN was independently associated with the occurrence of MACE (Table 3). In a second Cox regression model including established CMR prognosis markers (left ventricular ejection fraction, infarct size, microvascular obstruction), antecedent HTN was also independently associated with MACE after 12 months (hazard ratio: 2.55 [1.20–5.43], *p* = 0.02). 339 (43 %) of patients had HTN at the time of the acute event (defined as systolic blood pressure of ≥140 mmHg and/or diastolic blood pressure of ≥ 90 mmHg). In contrast with antecedent HTN, HTN at the time of the acute event was not significantly associated with the rate of MACE (log-rank *p* = 0.18).

**Table 2:** Event rates at 12 months after infarction

	All patients (*n* = 792)	Hypertension (*n* = 540)	No hypertension (*n* = 252)	*p* value
MACE, n (%)	53 (7)	45 (8)	8 (3)	<0.01
Death, n (%)	22 (3)	20 (4)	2 (1)	0.02
Re-infarction, n (%)	21 (3)	16 (3)	5 (2)	0.42
Heart failure event, n (%)	25 (3)	21 (4)	4 (2)	0.08

Data are given as number and percentage

*Abbreviations*: *MACE* major adverse cardiac events

**Table 3:** Predictors of MACE in uni- and multivariate Cox regression analysis

	Univariate		Multivariate	
	HR (95 % CI)	*p* value	HR (95 % CI)	*p* value
Age, >median	2.49 [1.38–4.47]	<0.01	-	-
Gender, 1 female	1.95 [1.12–3.40]	0.02	-	-
Smoking status, 1 yes	0.46 [0.24–0.87]	0.02	-	-
Diabetes mellitus, 1 yes	1.91 [1.07–3.41]	0.03	-	-
Hypertension, 1 yes	2.70 [1.27–5.72]	0.01	3.42 [1.45–8.08]	<0.01
Heart rate, >median	1.96 [1.12–3.43]	0.02	-	-
Killip-class, I-IV	1.90 [1.46–2.48]	<0.001	1.91 [1.40–2.60]	<0.001
Vessel Disease, I-III	1.34 [1.01–1.78]	0.04	-	-

Uni- and multivariate Cox regression analysis for the prediction of MACE at 12 months after infarction. Only significant variables in univariate analysis are reported

*Abbreviations*: *MACE* major adverse cardiac events, *HR* hazard ratio, *CI* confidence interval

### Antecedent hypertension and cardiovascular magnetic resonance findings

The median time between the index event and CMR scan was 3 [2–4] days. CMR findings and their relation with antecedent HTN are shown in Table 4. Patients with antecedent HTN had a similar extent of the area at risk (*p* = 0.18), infarct size (*p* = 0.74), microvascular obstruction (*p* = 0.93), and a comparable myocardial salvage index (0.20). There was also no significant difference in left ventricular ejection fraction between groups (*p* = 0.47). Antecedent HTN was not predictive for infarct size, microvascular obstruction, myocardial salvage index or left ventricular ejection fraction at linear regression analyses (all *p* > 0.05). The power of this study to exclude a difference in infarct size or LV ejection fraction of 3 % between groups is 94.9 and 98.5 %, respectively.

**Table 4:** Cardiovascular magnetic resonance findings

	All patients (*n* = 792)	Hypertension (*n* = 540)	No hypertension (*n* = 252)	*p* value
Area at risk, % LV	35 [25–48]	34 [25–47]	37 [26–49]	0.18
Infarct size, % LV	16.7 [8.5–24.9]	16.9 [8.8–24.8]	16.6 [8.3–25.0]	0.74
Presence of microvascular obstruction, n (%)	400 (51)	274 (51)	126 (50)	0.85
Extent of microvascular obstruction, % LV	0 [0–1.8]	0 [0.0–1.6]	0 [0.0–2.0]	0.93
Myocardial salvage index, % area at risk	51 [33–69]	50 [33–67]	54 [32–72]	0.20
LV ejection fraction, %	50 [43–58]	50 [43–58]	51 [44–58]	0.47
LV myocardial mass, g	132 [109–156]	134 [111–158]	131 [109–155]	0.38

Data are given as median plus interquartile range or number and percentage

*Abbreviations*: *CMR* cardiovascular magnetic resonance, *LV* left ventricular

## Discussion

This is the first study evaluating the impact of antecedent HTN on CMR parameters of myocardial salvage as well as myocardial and microvascular damage in patients undergoing PPCI for acute STEMI. There are two main findings: (1) antecedent HTN was independently associated with a higher rate of MACE and mortality at 12-month follow-up; (2) however, there were no differences in CMR-determined myocardial salvage, infarct size or extent of microvascular obstruction between patients with or without antecedent HTN. Together, these findings highlight the importance of a history of HTN as risk factor of adverse outcome in contemporary reperfused STEMI patients, which is however not attributed to differences in PPCI efficacy (myocardial salvage), extent of myocardial necrosis or microvascular injury as visualized by CMR.

### Antecedent hypertension, baseline risk profile and clinical outcome

As a consequence of its well established role as a major cardiovascular risk factor [1], antecedent HTN is a frequent finding in MI patients. In our analysis, history of HTN was present in 68 % of STEMI patients. These results fit well with recent prospective STEMI registry studies which reported a prevalence between 60 and 70 % [23, 24]. Antecedent HTN has been acknowledged as significant risk factor for adverse outcome in survivors of acute MI for many years [4–6]. For instance, Thune et al. found that antecedent HTN at the time of MI was strongly associated with an increased risk of future heart failure, stroke, cardiovascular death and a composite endpoint consisting of death, myocardial infarction, heart failure, stroke, or cardiac arrest in more than 14.000 patients included in the VALIANT trial (Valsartan in Myocardial Infarction Trial) [4]. Nevertheless, over the last years, reperfusion strategies and pharmacological interventions were significantly refined and consequently improved the outcome of patients presenting with acute MI [25]. These advances might have modified the relationship between HTN and the clinical course after STEMI [26]. Indeed, in a meta-analysis performed by Chen et al. the strength of the association between antecedent HTN and poor clinical outcome has decreased over time [11]. However, antecedent HTN was still associated with increased MACE and mortality rates at 12-month follow-up in our study. This was also observed after adjustment for confounding variables. Therefore, our findings highlight that even in the contemporary era of PPCI the simple information of HTN history can be used to identify patients at risk for adverse prognosis post-STEMI. This high risk group might also benefit from more intensified medical treatment strategies including aggressive antihypertensive treatment. Contrary to the information on antecedent HTN, HTN at the time of the acute event was not related with MACE in our study. Consequently, antecedent HTN seems of greater importance as compared with HTN at the time of STEMI.

It is well established that MI patients with a history of HTN show a distinctive baseline risk profile, irrespective of STEMI or non-STEMI presentation. Studies consistently observed that hypertensive patients with MI were older, more often female, had a higher body mass index, and higher rates of other cardiovascular risk factors including diabetes or hypercholesterolemia [6–10]. Our data confirm these previous findings and it is likely that these factors might at least partly explain the higher risk of hard clinical events of hypertensive STEMI patients. Further observations from our and other studies are the higher incidence of a previous cardiac disease and the greater extent of coronary artery disease [4, 7, 9, 10]. Since both have been proved to be associated with adverse clinical outcome in STEMI patients [27], these factors might also contribute to the increased risk of infarction patients with antecedent HTN. Recent studies could also disclose that diffuse myocardial fibrosis, measured by T1 mapping, is increased in patients with hypertension and previous infarction [28].

### Antecedent hypertension and infarct characteristics

It has been speculated that coronary occlusion in patients with pre-existing HTN might be associated with more extensive myocardial damage, greater myocardial dysfunction, and consequently adverse clinical outcome [11, 26]. Contrary, one might argue that a higher diastolic pressure could enhance coronary perfusion and collateral circulation with subsequently improved efficacy of reperfusion and limited expansion of myocardial necrosis. Studies investigating the impact of antecedent HTN on myocardial damage are very limited so far [29]. In particular, there is no study answering these questions by using the current reference standard technique, which is CMR [12]. CMR not only enables exact infarct sizing but also detailed tissue characterization of the jeopardized and infarcted myocardium [12]. These additional assessed parameters, primarily microvascular obstruction, but also myocardial salvage index provide strong prognostic information that is incremental to clinical, biomarker, electrocardiographic, and angiographic risk markers [15, 30]. To the best of our knowledge, the study by De Luca et al. [29]. is the first and only study that evaluated the impact of HTN on infarct size in 830 STEMI patients undergoing PPCI. The authors found that HTN was not associated with larger infarcts as determined by scintigraphy at 1 month after infarction. Nevertheless, some important limitations of this study have to be mentioned. The authors used a scintigraphic technique to assess infarct size, which is less accurate compared to CMR, especially for the detection of subendocardial infarcts [31]. Secondly, De Luca et al. were also not able to assess the association of antecedent HTN with myocardial salvage and microvascular dysfunction. Our study therefore complements and extends the previous literature by showing for the first time that the adverse outcome of reperfused STEMI patients with antecedent HTN is not associated with differences in reperfusion success or more pronounced myocardial injury.

### Limitations

Our study has some important limitations. Based on the in- and exclusion criteria of this multicenter clinical trial as well as the treatment of patients in specialized high-volume centers, our results might not be generalizable to all STEMI populations. Although AIDA STEMI was designed as all-comers trial to represent a “real-world” STEMI population [18, 20], further confirmation of our findings in other studies would be important. It is, however, important to note that the power of our study to exclude a 3 % difference in infarct size or LV ejection fraction between groups was approximately 95 %. Information on HTN was based on patient history and data on the duration of HTN as well as patients adherence to medication and effectiveness of HTN treatment, which could have prognostic implications as well, was not available. Nevertheless, our study shows that the simple assessment of antecedent HTN by anamnesis or during hospitalization has strong prognostic implications in STEMI patients treated with PPCI. Forty-eight patients (6 %) had a previous myocardial infarction. Chronic infarcts were not included in infarct size measurements and thus the “true volume” of the infarcted myocardium is not represented in the reported values. On the other hand, omitting those patients from statistical analysis did not significantly change the results.

## Conclusion

This study found higher rates of MACE and mortality in contemporary reperfused STEMI patients with antecedent HTN despite no difference in reperfusion success and extent of myocardial and microvascular damage as determined by CMR.

## Abbreviations

AIDA-STEMI: Abciximab Intracoronary versus intravenously Drug Application in ST-elevation myocardial infarction
CMR: Cardiovascular magnetic resonance
HTN: Arterial hypertension
LV: Left ventricular
MACE: Major adverse cardiac events
MI: Myocardial infarction
PPCI: Primary percutaneous coronary intervention
STEMI: ST-elevation myocardial infarction
VALIANT: Valsartan in Myocardial Infarction Trial
